# Melanoma in Chile: demographics and clinico-pathological features

**DOI:** 10.3389/fonc.2025.1604442

**Published:** 2025-09-09

**Authors:** Erica C. Koch Hein, Francisco Villanueva, Maysa Vilbert, Verónica Araya, Álvaro Abarzúa-Araya, Andrea Antúnez-Lay, Consuelo Cárdenas, Juan Camilo Castro, Francisco Dominguez, Katherine Droppelmann, Nicolás Droppelmann, Héctor Galindo, Augusto León, Jorge Madrid, Ximena Mimica, Montserrat Molgó, Sebastián Mondaca, Pablo H. Montero, Diego Romero, Pablo Uribe, Miguel A. Villaseca, Eugenio Vinés, Celeste Richardson, Cristian Navarrete-Dechent

**Affiliations:** ^1^ Department of Hematology and Oncology, School of Medicine, Pontificia Universidad Católica de Chile, Santiago, Chile; ^2^ Red de Salud UC CHRISTUS, Santiago, Chile; ^3^ Department of Dermatology, Faculty of Medicine, Universidad de Chile, Santiago, Chile; ^4^ Massachusetts General Hospital Cancer Center, Division of Hematology/Oncology, Department of Medicine, Boston, MA, United States; ^5^ School of Nursing, Pontificia Universidad Católica de Chile, Santiago, Chile; ^6^ Department of Dermatology, School of Medicine, Pontificia Universidad Católica de Chile, Santiago, Chile; ^7^ Department of Surgery, Complejo Asistencial Dr. Sótero del Río, Santiago, Chile; ^8^ Department of Surgical Oncology, School of Medicine, Pontificia Universidad Católica de Chile, Santiago, Chile; ^9^ Department of Surgery, Clínica Universidad de Los Andes, Santiago, Chile; ^10^ Fundación Arturo López Pérez, Santiago, Chile; ^11^ Department of Pathology, School of Medicine, Pontificia Universidad Católica de Chile, Santiago, Chile; ^12^ Department of Radiation Oncology, School of Medicine, Pontificia Universidad Católica de Chile, Santiago, Chile; ^13^ Medical Affairs Novartis Chile S.A, Santiago, Chile; ^14^ Millennium Institute for Intelligent Healthcare Engineering, Santiago, Chile

**Keywords:** melanoma, skin cancer, immunotherapy, diagnosis, survival, Latin America

## Abstract

**Background:**

Melanoma incidence is rising globally, yet epidemiological data from Latin America remain limited. In low- and middle-income countries, such data are essential for shaping evidence-based public health strategies.

**Objectives:**

To describe the demographic, clinical, and pathological characteristics of melanoma in Chile using a multi-institutional registry.

**Methods:**

We conducted a multicenter observational cohort study including patients ≥18 years with histologically confirmed melanoma diagnosed between 2014 and 2022 at one public and one private tertiary center in Santiago. Demographic, clinical, pathological, molecular, and survival data for cutaneous melanoma were analyzed using descriptive and survival statistics.

**Results:**

A total of 1,037 patients were included, of whom 979 (94.4%) had cutaneous melanoma. Among these patients, median age was 55 years and 54.8% were female. Cutaneous melanoma was more often diagnosed at early stages, particularly in the private setting. The most frequent histopathological subtypes were superficial spreading (31.6%), nodular (17.8%), and acral lentiginous melanoma (9.3%). Self-detection was the most common mode of identification (52.8%). Among patients with stage III–IV cutaneous melanoma tested for *BRAF*, 47.6% were positive. Higher risk of death was associated with advanced stage, nodular or amelanotic subtypes, *BRAF*-mutant tumors, male sex, and age ≥65 years. Only 34.8% of patients with stage IIB–IV cutaneous melanoma received systemic therapy.

**Conclusion:**

This study offers the most comprehensive characterization of melanoma in Chile to date, underscoring survival disparities by clinical, pathological, and healthcare access factors. Findings highlight the urgent need to expand access to early detection, molecular testing, and systemic therapies.

## 1 Introduction

Cutaneous melanoma (henceforth, “melanoma” unless otherwise specified) is a skin cancer derived from the skin melanocytes (1). Ultraviolet radiation (UVR) leads to DNA damage and genetic alterations in oncogenes such as *BRAF*, *NRAS*, *GNAQ*, *GNA11*, *c-kit*, and subsequent hits in *TERT*, *CDKN2A*, and *MITF*, among others (2). Melanoma cases are increasing, representing about 5% of all new cancers diagnosed in the U.S. (3). Worldwide, in the year 2020, the International Agency for Research on Cancer (IARC) reported 325,000 new cases and 57,000 people died from melanoma (4, 5). In the U.S., the incidence rate of melanoma was 21 per 100,000 new cases, and the death rate was 2.1 per 100,000 people per year between 2016 and 2020, according to the Surveillance, Epidemiology, and End Results (SEER) Program (3). Among the Hispanic people living in the U.S., the rates of new cases and deaths were lower, up to 3.8 and 0.7 per 100,000 people in 2020, respectively (6).

National data on the epidemiological profile of melanoma patients are scarce in Latin American countries (7), with most published data coming from small series or cohort studies (8). Chile does not have a National Registry of Cancer, although there are five-population-based provincial registries (9). None of these operate in the Santiago Metropolitan Region, which constitutes nearly half of the country´s population, and only three of these registries are integrated into the International Association of Cancer Registries (IACR). The incidence of melanoma in Chile varies significantly by the geographic location of its five registries, with an estimated average of 2.4 to 3 cases per 100,000 inhabitants (10).

Chile has a hybrid health system, with public health care FONASA (*Fondo Nacional de Salud*, or National Health Fund) serving the 70% of the population, mostly patients with lower socioeconomic status (SES) (11). Private health care is provided by ISAPREs (*Instituciones de Salud Previsional*), and other private health insurance providers. Notably, patients with private insurance are almost always treated at private healthcare institutions, while those covered by the public system primarily receive care at public institutions or choose to access private care by covering additional out-of-pocket costs.

Epidemiological data is especially relevant in low-middle-income countries for planning and implementing evidence- and cost-based public health policies. Also, this information is critical for educating the population and increasing awareness, which may lead to increased adherence to preventive measures and early disease detection. Therefore, aiming to fill-in this information gap and contribute to the growing literature of Latin America, we built a melanoma multicentric register in Chile. In this study, melanoma cases were classified into three major anatomical categories: cutaneous, mucosal, and ocular (including uveal and conjunctival types), following common clinical and epidemiological practice (12). Here, we present the first epidemiological analysis of a large Chilean melanoma registry cohort, describing the demographic, clinical, and pathological characteristics of the cutaneous subtype, as well as survival according to stage at diagnosis, sex, age, histopathological subtype, and *BRAF* mutation status.

## 2 Patients and methods

The study was approved by the Research Ethic Boards (REBs) of both institutions: the Pontificia Universidad Católica de Chile REB (ID 190812004), and the Hospital Dr. Sótero del Río REB (approval granted via official letter dated September 12, 2019; no formal ID number was issued, but the letter is available upon request).

### 2.1 Study design and data collection

In this multi-center observational retrospective and prospective cohort study, we included all patients of the age 18 or older with histopathological diagnosis of melanoma evaluated at the participating centers. Patient data was retrospectively collected at the Red de Salud UC Christus (Tertiary Care Academic private network), and Hospital Dr. Sótero del Río (Tertiary Care Public Center serving to a population of approximately 1,650,000 inhabitants) (13) between January 2014 and November 2019, and prospectively between December 2019 and June 2022. While patients from the public network are generally referred to the Hospital Dr. Sótero del Río based on their residential address, the private centers receive spontaneous consultations, often from patients residing in various districts of Santiago or from other regions of the country. Due to this open referral and access model—particularly in the private sector—this cohort does not represent a geographically defined population, and we therefore did not attempt to estimate melanoma incidence rates in this study.

The electronic biopsy records from the Pathology Department and the skin cancer tumor board documentation from each center were queried by melanoma diagnosis to identify eligible patients. A total of 323 variables were collected from electronic medical records by investigators and a research nurse, including demographic data, clinicopathologic features, treatment history, and follow-up information. The complete list of variables is available as **Supplementary Material**. Patients were anonymized using a study number. Database quality assessments were performed by an independent investigator comparing entered data with the electronic medical record source. The data cut-off for survival analysis was September 2022.

Our primary objective was to evaluate the demographic, clinical and pathologic characteristics of Chilean patients with cutaneous melanoma. Secondary objectives, also limited to cutaneous melanoma, included: (1) assessment of overall survival (OS) according to stage at diagnosis, based on the 8th edition of the American Joint Committee on Cancer (AJCC) Staging System for cutaneous melanoma (14); (2) assessment of OS according to clinical and molecular variables, including age, sex, histopathological subtype, and *BRAF* mutation status; (3) exploration of the method of melanoma detection; and (4) description of the use of systemic therapies, including immunotherapy, targeted therapy, and chemotherapy.

Although the WHO Classification of Tumours does not group mucosal melanomas into a single volume, these entities were described across different organ-specific classifications according to their anatomical site, including the Head and Neck Tumours (15), Urinary and Male Genital Tumors (16), Female Genital Tumours (17), and Digestive System Tumours (18). Uveal and conjunctival melanomas are included in the Eye Tumours volume (19), while cutaneous melanomas are covered in the Skin Tumours volume (20). For the purposes of this registry-based analysis, we adopted a unified classification framework to facilitate meaningful comparisons across melanoma subtypes. The histopathologic subtype of cutaneous melanoma was reported according to the *Protocol for the Examination of Biopsy Specimens from Patients with Invasive Melanoma of the Skin*, Version 1.1.0.0, published by the College of American Pathologists (CAP) (2025) (21).

The method of melanoma detection was classified based on the context in which the primary lesion was identified. *Self-detection* referred to cases where patients noticed a suspicious lesion themselves and consulted a physician specifically for this concern. *Incidental detection* occurred during a non-dermatologic medical consultation, in which the lesion was not the main reason for the visit but was noticed and referred for further evaluation. *Dermatology screening detection* typically took place in the context of preventive care or routine skin checks. Finally, *symptom-driven detection* referred to cases where the diagnosis followed a consultation prompted by symptoms related to primary or metastatic disease—such as lymphadenopathy, pain, or systemic symptoms—that ultimately led to further work-up and melanoma diagnosis.

### 2.2 Statistical analysis

Given that this was a population-based registry, we included all patients, and no formal sample size calculation was performed. Demographic and clinical characteristics of the cohort were analyzed using descriptive statistics. Categorical variables were presented as counts and percentages. Continuous variables were reported as mean ± standard deviation (SD) or median with interquartile range (IQR). Chi-square, Fisher’s exact, and Mann-Whitney U tests were used to assess differences in categorical and continuous variables among subgroups of interest. OS was defined as the time from the diagnosis to death from any cause, with the date obtained from death certificates. OS was censored at the date of last follow-up. Survival curves were estimated using the Kaplan–Meier method, and differences between groups based on pathological stage, sex, age group, *BRAF* mutation status, and histopathological subtype were assessed using the log-rank test. Histopathologic subtypes that reached median OS were included in the survival analysis (superficial spreading, nodular, acral lentiginous, and amelanotic melanomas). Lentigo maligna melanoma and ‘other’ subtypes were not included in this analysis. The median follow-up was estimated using the reversed Kaplan–Meier method. A Cox proportional hazards model was performed to perform a multivariable regression analysis to assess the association between stage, age, sex, histopathological subtype and *BRAF* mutation with the risk of mortality. Hazard ratios were adjusted for age and sex. All statistical tests were two-sided, and p value <0.05 was deemed significant. We performed all the statistical analysis in RStudio Version 4.0.2 (R Foundation for Statistical Computing, Vienna, Austria).

## 3 Results

### 3.1 Patients

A total of 1,037 patients were identified and included in the study. Demographic patient characteristics are reported in **Table 1**. The median age of the population was 55 (IQR: 18-97) years. We found a slight female predominance (54.8% *vs*. 45.2%, respectively). About 7.1% of patients reported a family history of melanoma in first or second-degree relatives.

**Table 1:** Demographic characteristics of 1,037 patients diagnosed with melanoma.

Characteristics	*n* (%)
Patients	1037
Sex	
Female	568 (54.8)
Male	469 (45.2)
Age (years), median (IQR)	55 (42-68)
Health Care Provider	
Private (ISAPREs)	431 (41.6)
Public (FONASA)	413 (39.8)
Other private health insurance	57 (5.5)
Unknown	136 (13.1)
Center of Diagnosis	
Private (Red de Salud UC Christus)	822 (79.3)
Public (Hospital Dr. Sótero del Río)	215 (20.7)

IQR, Interquartile range; ISAPRE, Instituciones de Salud Previsional; FONASA, Fondo Nacional de Salud.

### 3.2 Clinical and pathologic characteristics

Of the 1,037 patients included in the registry, the majority had cutaneous melanoma (n = 979, 94.4%), followed by mucosal (n = 29, 2.8%), ocular (n = 19, 1.8%), and unknown primary location (n = 10, 1.0%). **Table 2** summarizes the clinical and pathological characteristics of patients with cutaneous melanoma.

**Table 2:** Clinical and pathologic features of 979 patients diagnosed with cutaneous melanoma.

Characteristics	*n* (%)
Histopathological diagnosis	
Invasive	674 (68.8)
* In situ*	305 (31.2)
Anatomic primary site	
*Single*	
Lower extremities	314 (32)
Trunk	259 (26.5)
Head and neck*	193 (19.8)
Upper extremities	167 (17.1)
Genital	4 (0.4)
Unknown	27 (2.8)
*Synchronous* ^†^	15 (1.5)
Method of Detection	
Self-detection	517 (52.8)
Incidental detection	108 (11)
Dermatology screening	99 (10.1)
Locoregional or metastasis symptoms	32 (3.3)
Missing	223 (22.7)

^*^Excludes mucosal or ocular melanomas.

^†^Synchronous melanoma was observed in the following anatomical combinations: head/neck with trunk (n = 3), head/neck with upper limb (n = 1), two primaries on the trunk (n = 2), trunk with upper limb (n = 2), trunk with lower limb (n = 3), and upper limbs with lower limbs (n = 4).

Melanoma *in situ* was diagnosed in 305 (31.2%) patients, and invasive melanoma in 674 (68.8%) patients. Among the female patients, 67.2% presented with invasive cutaneous melanoma, while the corresponding percentage for male patients was 70.9% (p = 0.22). Further stratification by age revealed that for individuals aged 65 and older, 70.9% presented with invasive cutaneous melanoma, similar as for those below 65 years, for whom the percentage was 68% (p = 0.37).

The predominant primary sites for cutaneous melanoma were the lower extremities (32%) and the trunk (26.5%). Among females, the lower extremities were the most common site of origin (38.9%), whereas in males, melanomas most often arose on the trunk (33.8%) (p < 0.001). In all, 52.8% (n=517) of patients had their cutaneous melanoma diagnosed through self-detection and 10.1% (n=99) of patients through formal screening during a dermatologist visit (**Table 2**). The percentage of cutaneous melanomas diagnosed through dermatology screening varied between private (16.6%) and public healthcare (3.6%) (p < 0.001).

Regarding invasive cutaneous melanoma (n = 674), the most prevalent histopathologic subtypes were superficial spreading (31.6%), nodular (17.8%), and acral lentiginous (9.3%) (**Table 3**). There were no statistically significant differences in the distribution of histopathologic subtypes by sex (p = 0.59) or by age group (≥65 *vs*. <65 years; p = 0.09).

**Table 3:** Histopathological subtype for 674 invasive cutaneous melanomas according to CAP Protocol v1.1.0.0 (2025) (21).

Histopathological subtype	*n* (%)
Superficial spreading	213 (31.6)
Nodular	120 (17.8)
Acral	63 (9.3)
Lentigo maligna melanoma	24 (3.6)
Amelanotic	11 (1.6)
Other*	18 (2.7)
Missing	225 (33.4)

*Other histopathological subtypes: desmoplastic melanoma, mixed desmoplastic/non-desmoplastic melanoma, spitzoid melanoma, melanoma arising in a giant congenital nevus, melanoma arising in a blue nevus, nevoid melanoma, dermal melanoma, melanoma not otherwise specified (NOS).

Among the 29 patients with mucosal melanoma in our cohort, the most frequent primary sites were the gastrointestinal tract (n = 10) and the female genital tract (n = 9). Within the gastrointestinal group, tumors were located in the esophagus (n = 1), stomach (n = 1), anal canal (n = 2), and rectum (n = 5). Head and neck mucosal melanomas accounted for eight cases, including tumors in the nasal cavity and paranasal sinuses (n = 5), oral cavity (n = 2), and nasopharynx (n = 1). One patient had a primary tumor in the urinary tract, specifically the urethra. In two cases, the primary mucosal site of origin could not be determined from the available records and was therefore classified as unknown.

Of the 19 patients with ocular melanoma, 14 were classified as uveal, including 11 choroidal cases and three of unknown uveal subtype. Four patients had conjunctival melanoma. In one case, the specific ocular subtype (uveal or conjunctival) could not be determined and was recorded as unspecified.

#### 3.2.1 Staging details at diagnosis for cutaneous melanoma

Among patients with cutaneous melanoma (N = 979), 62.1% were diagnosed at early stages (pathologic stage 0 or stage I). As shown in **Table 4**, melanoma was more frequently diagnosed as localized disease (*in situ*, stage I or II) for patients seen in the private healthcare institution (73.4%) compared with those diagnosed at the public institution (61.9%) (p < 0.001).

**Table 4:** Pathological stage of 979 patients with cutaneous melanoma at presentation, according to the AJCC 8^th^ edition (14).

Pathological stage	All (*n*=979)	Public (*n*=202)	Private (*n*=777)	*p*-value
	*n* (%)	*n* (%)	*n* (%)	
0	305 (31.2)	42 (20.8)	263 (33.8)	<0.01*
I	303 (30.9)	55 (27.2)	248 (31.9)	
II	87 (8.9)	28 (13.9)	59 (7.6)	
III	116 (11.8)	35 (17.3)	81 (10.4)	
IV	51 (5.2)	20 (9.9)	31 (4.0)	
Missing^†^	117 (12.0)	22 (10.9)	95 (12.2)	

*p-value corresponds to comparison of stages 0–II *vs* III–IV between public and private systems.

^†^Missing does not include *in situ* cases.

For the patients with cutaneous melanoma, 51 patients (5.2%) presented with distant metastasis at diagnosis. The most frequent sites of distant metastasis were the lung, non-regional lymph nodes and visceral other than lung, in 70.6%, 62.8% and 62.8%, respectively (**Table 5**).

**Table 5:** Sites of distant metastasis at diagnosis in 51 patients with stage IV cutaneous melanoma.

Site*	*n* (%)
Lung	36 (70.6)
Non-regional lymph node	32 (62.8)
Visceral other than lung ^†^	32 (62.8)
Bone	26 (50.9)
Central nervous system	22 (43.1)
Soft tissue	19 (37.3)
Skin	6 (11.8)
Mucosa	1 (1.9)

*Categories are not mutually exclusive.

^†^Includes visceral metastases to organs such as liver, gastrointestinal tract, or kidneys.

#### 3.2.2 Molecular analysis in patients with advanced cutaneous melanoma

*BRAF* mutation analysis was performed in 84 patients with stage III or IV cutaneous melanoma (**Table 6**). *BRAF* mutation was detected in 47.6% of the 84 patients with stage III or IV melanoma that underwent this molecular analysis. The demographic and clinical features of these patients harboring *BRAF* mutation are described in **Table 7**.

**Table 6:** *BRAF* analysis in 84 patients with stage III or IV cutaneous melanoma.

*BRAF* analysis	*n* (%)
*BRAF* status*	
Mutant	40 (47.6)
Wild type	44 (52.4)
*BRAF* mutation^†^	
V600 (not specified)	26 (65.0)
V600E	11 (27.5)
V600K	1 (2.5)
Not reported	2 (5.0)

(*) Calculated from the 84 patients who underwent molecular testing for *BRAF*.

(^†^) Calculated from the 40 patients with identified *BRAF* mutation.

**Table 7:** Demographic and clinical features of 40 patients with stage III or IV cutaneous melanoma harboring *BRAF* mutation.

Characteristics	*n* (%)
Sex	
Female	18 (45.0)
Male	22 (55.0)
Age (years), median (IQR)	51 (34.3-61.8)
Histological subtype	
Nodular	16 (40.0)
Superficial spreading	4 (10.0)
Amelanotic	3 (7.5)
Epithelioid	1 (2.5)
Missing	16 (40.0)
Anatomic primary site	
Head and neck (excluding ocular)	6 (15.0)
Trunk	11 (27.5)
Upper extremities	8 (20)
Lower extremities	7 (17.5)
Synchronous	1 (2.5)
Unknown	7 (17.5)

IQR, Interquartile range.

*NRAS* mutation analysis was tested in 25 patients with advanced melanoma; seven of them were positive for Q61X mutation. Only four patients with advanced melanoma were tested for *KIT* mutations, with one of them resulting positive for mutation E554K.

### 3.3 Treatment modalities used for invasive cutaneous melanoma

A total of 86.1% of the 674 patients underwent surgery with curative intent. Radiation therapy (RT) was administered to 65 patients (9.7% of the cohort); among them, 29 (44.6%) received RT as adjuvant treatment, 28 (43.1%) as palliative treatment, and eight (12.3%) as definitive therapy. Of the 221 patients with invasive cutaneous melanoma stage IIB or higher, only 77 (34.8%) received systemic therapy (**Table 8**).

**Table 8:** Systemic therapy in 77 patients with stage IIb or higher cutaneous melanoma.

Therapeutic intent	Adjuvant (*n*=47)	Palliative (*n*=30)	Total (%) (*n*=77)
Immunotherapy	46	24	70 (90.9)
Anti-PD-1*	45	19	64 (83.1)
Anti-CTLA-4**	0	2	2 (2.6)
Combined^†^	0	2	2 (2.6)
Unknown	0	1	1 (1.3)
Targeted therapy^††^	1	4	5 (6.5)
Chemotherapy‡	0	2	2(2.6)

*Nivolumab or pembrolizumab; **Ipilimumab; ^†^Nivolumab/Ipilimumab; ^††^Combined anti-BRAF and anti-MEK (Dabrafenib/Trametinib or Vemurafenib/Cobimetinib); ^‡^Temozolomide or Paclitaxel/Carboplatin.

Among these, 61% initiated treatment in the adjuvant setting. Most of them (97.9%) received immunotherapy, while one patient (2.1%) received targeted therapy. The majority of these adjuvant-treated patients (n = 41, 89%) had stage III disease. The remainder included one patient (2.2%) with completely resected stage IV disease, two patients (4.4%) with stage IIB, and one patient (2.2%) with stage IIC; staging data were missing for one patient. Of the 77 patients who received systemic therapy, 39% were treated for unresectable or metastatic disease. Among them, 80% received immunotherapy, 13.3% received targeted therapy, and 6.7% received chemotherapy. The one patient with missing immunotherapy details in the palliative setting was treated at another institution covered by their health insurance; while clinical notes confirmed immunotherapy was administered, they did not specify whether it was anti–PD-1 monotherapy or combined with anti–CTLA-4.

### 3.4 Survival for invasive cutaneous melanoma

**Figure 1:**
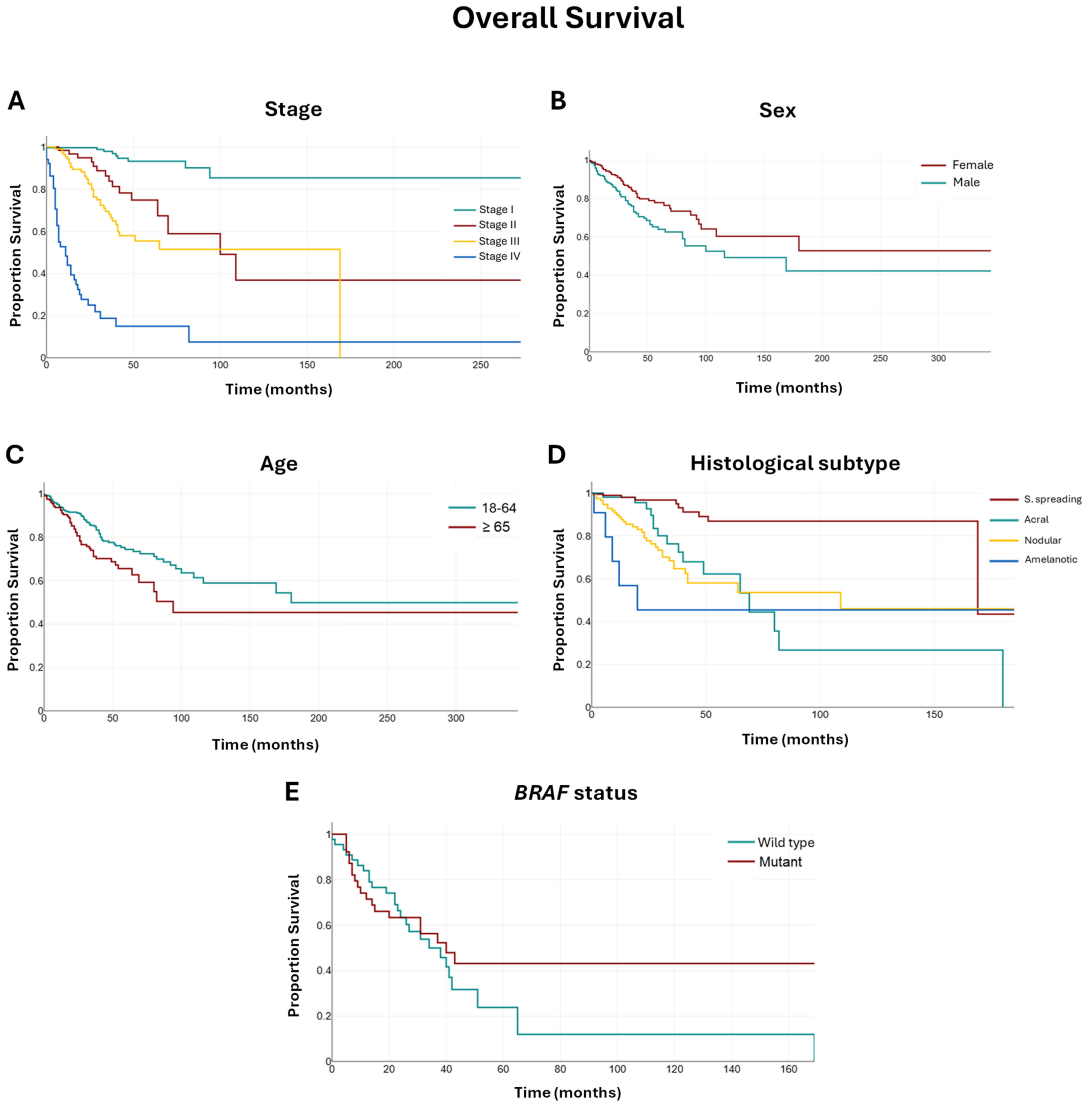
Kaplan–Meier survival curves **(A–E)** according to pathological stage, sex, age group, histopathological subtype, and *BRAF* mutation status in patients with invasive cutaneous melanoma. Abbreviations: S. spreading: superficial spreading melanoma. Note: Lentigo maligna melanoma and other subtypes were not included in this analysis.

Kaplan–Meier analysis revealed significant differences in overall survival (OS) according to pathological stage, sex, age group, and histopathological subtype, but not by *BRAF* mutation status (**Figure 1**). The median follow-up for the cohort was 31 months.

Patients with stage I disease had the longest OS, with the median not reached during the observation period. In contrast, median OS declined with advancing stage: 100 months for stage II (95% CI: 64–109), 69 months for stage III (95% CI: 41–65), and 11 months for stage IV (95% CI: 6–16).

When stratified by sex, females had a longer median OS compared with males (median not reached *vs*. 116 months; 95% CI: 80–169). Similarly, patients aged 18–64 years showed a significantly longer median OS of 180 months (95% CI: 116–180) compared with 94 months (95% CI: 69–94) in those aged ≥65 years.

Regarding histopathological subtypes, patients with superficial spreading melanoma had the longest median OS at 169 months (95% CI: 169–169), followed by nodular melanoma (109 months; 95% CI: 42–109), acral lentiginous melanoma (69 months; 95% CI: 49–82), and amelanotic melanoma (20 months; 95% CI: 9–20).

The presence of a *BRAF* mutation was not associated with a statistically significant difference in OS: median OS was 40 months (95% CI: 20–43) for patients with *BRAF*-mutant tumors *versus* 34 months (95% CI: 24–41) for those with *BRAF* wild-type tumors.

In the multivariable Cox regression model, pathological stage remained the strongest predictor of all-cause mortality. Compared with patients with stage I disease, the adjusted hazard of death increased significantly in those with stage II, stage III, and was highest in stage IV (**Table 9**).

**Table 9:** Adjusted hazard ratios for all-cause mortality in patients with invasive cutaneous melanoma.

Variable	Patients (*n*)	Events *(n)*	HR*	(95% CI)	*p*-value
Stage					
I	303	9	1	(reference)	
II	87	15	2.3	(1.24–4.27)	0.008
III	116	37	4	(2.48–6.48)	<0.001
IV	51	40	21.1	(12.78-34.84)	<0.001
Sex					
Male	309	71	1	(reference)	
Female	365	54	0.63	(0.44-0.89)	0.01
Age (years)					
18-64	467	75	1	(reference)	
≥ 65	207	50	1.56^†^	(1.09-2.25)	0.015
**Subtype**					
Superficial spreading	213	10	1	(reference)	
Nodular	120	34	2.18	(1.43–3.32)	<0.001
Acral	63	16	1.69	(0.97–2.94)	0.065
LMM	24	3	0.48	(0.15–1.56)	0.223
Amelanotic	11	5	3.53	(1.41–8.84)	0.007
Other	18	4	2.02	(0.72–5.71)	0.184
*BRAF* status					
Wild type	44	26	1	(reference)	
Mutant	40	19	3.05	(1.86–5.01)	<0.001

*Adjusted for age (continuous) and sex. ^†^ Adjusted for sex. HR, Hazard Ratio; LMM, Lentigo maligna melanoma.

Female sex was independently associated with a lower risk of death compared to male sex, whereas age ≥65 years was linked to increased mortality. Regarding histopathological subtype, nodular melanoma conferred a significantly higher adjusted mortality risk relative to superficial spreading melanoma, and amelanotic melanoma was also associated with increased risk. No statistically significant differences were observed for acral lentiginous melanoma, lentigo maligna melanoma, or other melanoma subtypes (**Table 9**). Finally, the presence of a *BRAF* mutation was associated with a higher adjusted risk of death compared to wild-type cases (**Table 9**).

## 4 Discussion

In this large cohort of 1,037 melanoma patients from two tertiary care centers in Chile, we provide a comprehensive characterization of the clinical and pathological features of melanoma in a real-world Latin American setting. We identified several key findings: (1) most patients with cutaneous melanoma were diagnosed at an early stage, particularly in the private healthcare setting; (2) self-detection was the most common method of lesion identification; (3) superficial spreading melanoma was the most frequent histopathological subtype, followed by nodular and acral lentiginous melanoma; (4) overall survival and risk of death differed significantly across subgroups, with statistically worse outcomes among patients with advanced stage at diagnosis, nodular or amelanotic melanoma, older age, and male sex. Additionally, the presence of *BRAF* mutations was associated with an increased risk of mortality; and (5) a limited proportion of patients with advanced disease received systemic therapy.

The mean age at diagnosis around the sixth decade of life is similar to worldwide data (22), other registries from Latin America (23–26), and prior studies from Chile (27, 28). The female predominance observed in our cohort aligns with findings from other retrospective Chilean studies of patients with cutaneous malignant melanoma, where the proportion of female patients ranged from 60.6% to 64.9% (27–30). Similarly, two Brazilian studies reported that 56.3% to 58.8% of cutaneous melanoma cases occurred in women (31, 32). This pattern is also reflected in a Colombian population-based registry (60%) (26) and a Mexican study (58.5%) (25), whereas male predominance was reported in Argentinian, North American, and global datasets (5, 24, 33–35). In Europe, the incidence of cutaneous melanoma is also generally higher among women (36). These differences might be associated with biological, ethnic, occupational, and cultural elements that vary between female and male patients in distinct countries and cultures.

The distribution of primary tumor site in our cohort showed a statistically significant difference by sex, with melanomas more frequently located on the lower extremities in women and on the trunk in men (p<0.001). This anatomical distribution has been consistently reported in previous studies and is thought to reflect differences in patterns of sun exposure, clothing, and behavioral factors (22, 23, 37–42).

It is noteworthy that a substantial portion of cases, specifically 51.5%, of the diagnosed melanoma cases stemmed from patients’ self-awareness. This underscores the pivotal role of individual vigilance in early detection and emphasizes the critical need for widespread public education initiatives at different population levels (e.g. primary education, involvement of laypersons such as hairdressers and podiatrists, etc.). Similar results have been obtained in prior studies in which melanoma self-detection was the primary method of melanoma diagnosis, with 47%-57% of patients detecting their own melanoma (43–45). By enhancing the knowledge and awareness of melanoma signs and encouraging proactive self-examinations, we can potentially contribute to early detection, timely intervention, and improved outcomes. This emphasizes the significance of educational campaigns aimed at empowering the population to take an active role in their skin health. The markedly lower proportion of cutaneous melanomas diagnosed through dermatology screening in the public sector (3.6%) compared to the private sector (16.6%) (p < 0.001) highlights disparities in access to preventive care, consistent with previous studies reporting that individuals with higher socioeconomic status are more likely to seek dermatologist evaluations and present with earlier-stage disease (45–47). Additionally, 10.7% of melanoma cases were incidentally identified during medical visits originally scheduled for reasons unrelated to skin concerns. Although with a more controversial role, population-based screening by dermatologists, general physicians, or advanced medical providers might also have a critical role in the early detection of melanoma and potential reduction in mortality in selected patients (48, 49).

The higher prevalence of invasive melanoma compared to *in situ* cases may be attributed to the fact that patients with more advanced disease, requiring a multidisciplinary treatment approach, are often referred to our tertiary centers. Therefore, this might not reflect the true proportion of melanoma *in situ vs*. invasive cases in our country.

Similar to findings in Caucasian populations, we noted a predominance of the superficial spreading melanoma subtype, with nodular melanoma following as the second most frequent subtype (50). Interestingly, acral was the third most common histopathogical subtype. Higher proportion of acral subtype has been reported in other series among Hispanic White and Latino population (25, 51–53). A recent Mexican study including 1219 patients reported that 44% of their cases were acral melanoma (25). Furthermore, a strong relationship between the frequency of acral melanoma subtype and the percentage of people with mixed Spanish and Amerindian ancestry was described in a Peruvian cohort (54). Unfortunately, 34.2% of patients (N=230) in our cohort had no histopathological subtype information, often due to prior excisional biopsies performed externally, with pathology reports lacking subtype data.

As in other countries, significant differences in population and disease characteristics are observed in Chile regarding public and private healthcare systems, reflecting underlying socioeconomic disparities (7, 24, 46, 55–57). In our cohort, patients diagnosed in the public institution had significantly more advanced disease, with 26.7% presenting with stage III or IV melanoma compared to 14.4% in the private institution (p < 0.001). This is consistent with findings from one of the largest Chilean retrospective cohorts, which reported higher rates of invasive melanoma and Breslow thickness >1 mm among patients treated in the public setting (27). Moreover, the proportion of patients presenting with metastatic disease in the public setting (9.9%) in our cohort exceeded that reported in the U.S. SEER database (4%) (3, 58). Socioeconomic disparities have been consistently associated with later-stage melanoma diagnosis. Lower education levels are linked to decreased awareness and fewer skin examinations (59–62), and low SES remains an independent predictor of advanced disease even after adjusting for education (63). Limited access to dermatologic care in low-SES populations further contributes to diagnostic delays (64). In Chile, where the public healthcare system primarily serves lower-SES groups, these factors likely explain the higher burden of advanced and metastatic melanoma observed in that setting.

Consistent with international melanoma guidelines, the primary therapeutic approach in our cohort was surgery. A minority of patients underwent adjuvant systemic therapy. A relevant finding of our series was that nearly half of patients diagnosed with advanced cutaneous melanoma (stages III and IV) did not undergo *BRAF* testing. *BRAF* testing is considered the standard practice for determining the optimal systemic therapy approach in both adjuvant and metastatic scenarios, as outlined in established international guidelines (65, 66). Systemic therapy with immune checkpoint inhibitors or combined BRAF/MEK-targeted therapy is recommended by international guidelines for patients with resected stage IIB to IV melanoma, as well as for those with unresectable or metastatic disease (65, 66). However, in our cohort, only a minority of patients received these treatments. Among the 221 patients with stage IIB or higher melanoma, only 34.8% received systemic therapy—either as adjuvant treatment or as first-line therapy for advanced, unresectable, or metastatic disease. This highlights a significant gap between guideline-based standards of care and real-world clinical practice in our setting, likely driven by limited drug availability, delayed regulatory approvals, and coverage restrictions in the public healthcare system. In Chile, anti-PD-1 immune checkpoint inhibitors have only been available and reimbursed in the public healthcare institutions since 2019 for patients with melanoma in the adjuvant or metastatic setting. Other recommended therapies, such as anti-CTLA-4 antibodies (e.g., ipilimumab) and BRAF/MEK inhibitors, remain unavailable through the public health system.

In our cohort, *BRAF* mutations were identified in 47.6% of patients with stage III or IV cutaneous melanoma who underwent molecular testing, a frequency consistent with global estimates ranging from 40% to 60% (67–70). As in previous reports, the most frequent mutation detected was in codon V600 (68). However, due to test limitations, 65% of cases were reported only as “V600” without specifying the exact variant; among those with detailed results, V600E was the most common (27.5%), followed by V600K (2.5%), consistent with international data where V600E accounts for the majority of *BRAF*-mutated melanomas (68, 71). Patients harboring *BRAF* mutations were younger (median age 51 years), predominantly male, and most frequently were diagnosed with nodular melanoma subtype—a pattern that aligns with previous cohorts where nodular melanoma is commonly associated with *BRAF* mutations (68, 71). The trunk was the most frequent anatomical site in our series (27.5%), consistent with previous reports in *BRAF*-mutant melanoma where this location predominates over the extremities and head and neck (68, 71, 72). Notably, the presence of a *BRAF* mutation was not associated with a statistically significant difference in unadjusted overall survival. However, in multivariable analysis, *BRAF* mutation conferred a significantly increased risk of death (HR: 3.05; 95% CI: 1.86–5.01), echoing findings from historical pre-targeted therapy cohorts with median survival was eight to ten months (73). For instance, Long et al. reported a median OS of 11.1 months in untreated *BRAF-*mutant metastatic melanoma, compared with 46.1 months in *BRAF* wild-type patients (68). In our cohort, only four patients received BRAF/MEK inhibitors in the palliative setting, reinforcing the continued limitations in access to precision oncology. While our sample size was limited, this is, to our knowledge, the first Chilean study to report on *BRAF* mutation prevalence and clinical correlations in melanoma. As highlighted by Salman et al. in a recent regional review, molecular epidemiologic data on melanoma remain scarce in Latin America, with only a handful of small series from Argentina, Mexico, and Brazil reporting similar mutation frequencies (74). Our findings contribute to filling this knowledge gap and emphasize the need to expand access to molecular diagnostics and targeted therapies, particularly in public healthcare systems across the region.

### 4.1 Limitations

Limitations include the observational nature of the study, and the relatively short follow-up period. We did not centrally review all biopsies performed outside our institution, contributing to variability in pathology interpretation across different pathologists and to missing data. It is well known that interobserver concordance rates among pathologists are low for melanocytic lesions, including invasive melanomas (75).

As with many retrospective registry-based studies, some clinical variables were missing due to incomplete or inconsistent documentation in medical records. For example, skin characteristics such as Fitzpatrick skin type and the presence of multiple or atypical moles were not reported for most patients due to missing data.

In addition, insurance status was unknown in 136 patients, as this information is not automatically recorded in the electronic health records of the participating centers and relies on manual physician input. In 27 cases, the primary tumor location was undocumented, often because biopsies or diagnostic workups were performed at external institutions and original reports were unavailable. Data on the method of melanoma detection were missing in 223 cases, reflecting inconsistencies in the recording of clinical history. Furthermore, 225 patients had unknown histopathologic subtypes, typically due to inaccessible or insufficient pathology reports from external institutions. These limitations underscore the challenges of retrospective data collection in large multicenter cohorts.

## 5 Conclusions

This study provides an overview of melanoma at presentation and offers insights into its initial management across two markedly different healthcare settings in Chile. By establishing and maintaining a dedicated registry, we aim to generate essential data that can inform evidence-based policymaking and support a more strategic, targeted approach to addressing the evolving cancer landscape in the country.

This registry is envisioned to be an ongoing resource that can enhance our understanding, inform public health initiatives, and support advancements in melanoma care.

## Data Availability

The raw data supporting the conclusions of this article will be made available by the authors, without undue reservation.
